# Involvement of the larynx motor area in singing-voice perception: a TMS study^†^

**DOI:** 10.3389/fpsyg.2013.00418

**Published:** 2013-07-11

**Authors:** Yohana Lévêque, Neil Muggleton, Lauren Stewart, Daniele Schön

**Affiliations:** ^1^Laboratoire Parole et Langage, Centre National de la Recherche Scientifique and Aix-Marseille UniversitéAix-en-Provence, France; ^2^Institute of Cognitive Neuroscience, University College LondonLondon, UK; ^3^Music, Mind and Brain, Department of PsychologyGoldsmiths, London, UK; ^4^Institut de Neurosciences Cognitives de la Méditerranée, Université de la MéditerranéeMarseille, France

**Keywords:** auditory–motor interactions, singing, larynx motor representation, TMS, voice perception

## Abstract

Recent evidence has reported that the motor system has a role in speech or emotional vocalization discrimination. In the present study we investigated the involvement of the larynx motor representation in singing perception. Twenty-one non-musicians listened to short tones sung by a human voice or played by a machine and performed a categorization task. Thereafter continuous theta-burst transcranial magnetic stimulation was applied over the right larynx premotor area or on the vertex and the test administered again. Overall, reaction times (RTs) were shorter after stimulation over both sites. Nonetheless and most importantly, RTs became longer for sung than for “machine” sounds after stimulation on the larynx area. This effect suggests that the right premotor region is functionally involved in singing perception and that sound humanness modulates motor resonance.

## INTRODUCTION

Since the discovery of auditory–visual mirror neurons in the macaque ventral premotor cortex (Kohler et al., 2002), neuroimaging studies in humans have provided evidence of motor/premotor activations during speech listening (see Aglioti and Pazzaglia, 2010, for a review) as well as during listening to other human-produced sounds (e.g., Chang et al., 2009). This mirror-like motor activity reflects the precise somatotopy of orofacial representations in the primary motor cortex. In particular, an increased excitability of the tongue muscles was reported when the tongue representation in motor cortex was stimulated by transcranial magnetic stimulation (TMS; Fadiga et al., 2002). The TMS study conducted by D’Ausilio et al. (2009) confirmed the precision of this somatotopy, showing distinct effects on perceptual performance when stimulating lip and tongue representations.

In the speech domain, a number of researchers have considered these motor activations as being necessary for speech processing and understanding (Iacoboni, 2008), in line with the suggestions of Liberman et al. (1967) that speech is perceived by mapping sounds into articulatory gestures. However, the exact functional role of this motor activity is still debated (Scott et al., 2009), with some studies arguing that sensorimotor information is not required in most contexts of speech perception (Hickok, 2009). To investigate this question, the use of TMS as an interference technique offers a unique opportunity. It allows the researcher to test the causal relationship between motor activity in perception and performance by disrupting the function of a target area in the premotor or motor cortex. TMS has thus been used to alter activity of the orofacial premotor zone while participants were performing phonologic tasks such as phoneme discrimination (Meister et al., 2007), phoneme categorization (Möttönen and Watkins, 2009) or phoneme segmentation (Sato et al., 2009). In each case, results revealed a reduction in performance after stimulation, suggesting that this orofacial motor zone plays a necessary role in the processing of speech, at least in the framework of phonological tasks. In the same vein, two recent TMS studies have suggested that the larynx premotor region may be involved in discrimination tasks with non-linguistic vocal stimuli. After TMS on the premotor cortex, D’Ausilio et al. (2011) found a reduction in response times in a discrimination task with pitch-shifted vowel utterances while Banissy et al. (2010) found an increase in response times in an emotion discrimination task following continuous theta-burst stimulation.

In the present study we investigated the involvement of the larynx premotor area in a task requiring listeners to identify whether a heard stimulus was generated by a human voice or a machine. We hypothesized that the perception of a human-produced sound like the singing voice would induce motor resonance via interactions between the auditory and vocal systems. In line with this hypothesis we predicted that perceptual processing of sung tones would be disrupted by stimulation of the larynx premotor area. As control sounds, we used voice sounds distorted by saturation. As the control sounds could not be mapped onto bodily representations, we hypothesized that they would not be affected by the larynx area functional disruption.

## MATERIALS AND METHODS

### PARTICIPANTS

Twenty-one healthy right-handed participants (12 females) took part in the experiment (mean age: 25.4; SD: 4.4, average music training: 1.3 years; SD: 2.6) after giving informed consent. The experiment was approved by the local ethics committee.

### STIMULI

**FIGURE 1:**
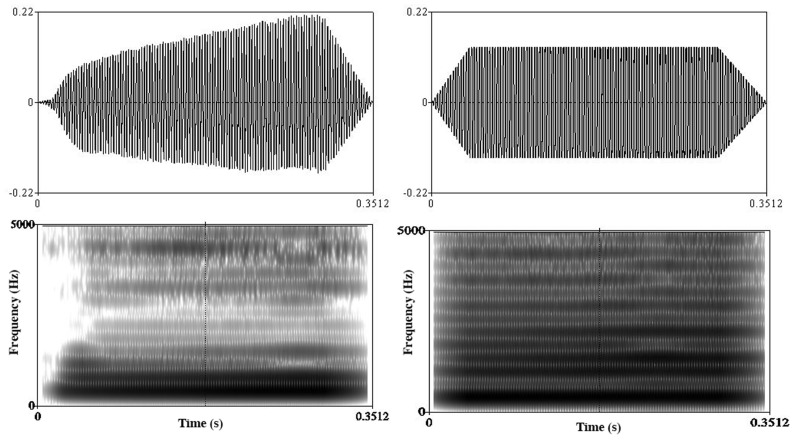
**Waveforms (top) and spectrogram (bottom) of a natural voice stimulus (left) and a “machine” stimulus (right)**.

Natural voice stimuli were recorded in an anechoic room by a female singer wearing a headset with microphone (Sennheiser PC131). Each stimulus consisted of a single pitch ranging from E3 to B5 sung on the vowel [o], without vibrato. In order to give the stimuli a non-human quality for the “machine” condition, the natural sounds were distorted with a fuzz-like saturation effect using the sound editor Adobe Audition (**Figure 1**). All stimuli were cut at 350 ms applying a 50 ms fade out and were normalized in intensity using an equal loudness contour.

### PROCEDURE

An offline TMS paradigm was used to compare performance on the same task before and after continuous theta-burst TMS had been given (see Transcranial Magnetic Stimulation).

Stimuli were delivered aurally through headphones. Participants were instructed to make a speeded response, indicating whether each presented sound was produced by a voice or a machine, by pressing the “V” or “M” button, with their right forefinger and middle finger. The position of M and V buttons was counter-balanced across subjects. The following stimulus was presented 1150 ms after the response. Participants were first familiarized with the experimental procedure by completing five training trials. They then performed three experimental runs (baseline), each including 46 trials per condition, presented in a random order, for a total of 276 trials (task length: 9 min). Reaction times (RTs) were recorded using E-prime with respect to stimulus offset (Psychology Software Tools, Inc.).

Thereafter, while they were at rest, 11 participants received repetitive TMS over the vertex (control group), and 10 over the right larynx premotor area (experimental group) after identification of the target location on the scalp (see Transcranial Magnetic Stimulation). The vertex was determined as the midpoint between inion and nasion and between left and right tragus. The coordinates of the right premotor (PM) larynx site were based on Talairach coordinates reported by Brown et al. (2008) in a task of glottal sound production and vocalization (*x* = 53; *y* = 4; *z* = 42)^1^. A T1-weighted magnetic resonance imaging (MRI) structural scan was used to localize this site in each subject. Brown’s coordinates were converted to Brainsight coordinate space by the linear transformation implemented in Brainsight. The right hemisphere stimulation was also motivated by previous studies suggesting right hemisphere dominance during singing (Perry et al., 1999; Riecker et al., 2000; Jeffries et al., 2003). After a 5 min rest period, all the participants were asked to perform the categorization task a second time.

### TRANSCRANIAL MAGNETIC STIMULATION

**FIGURE 2:**
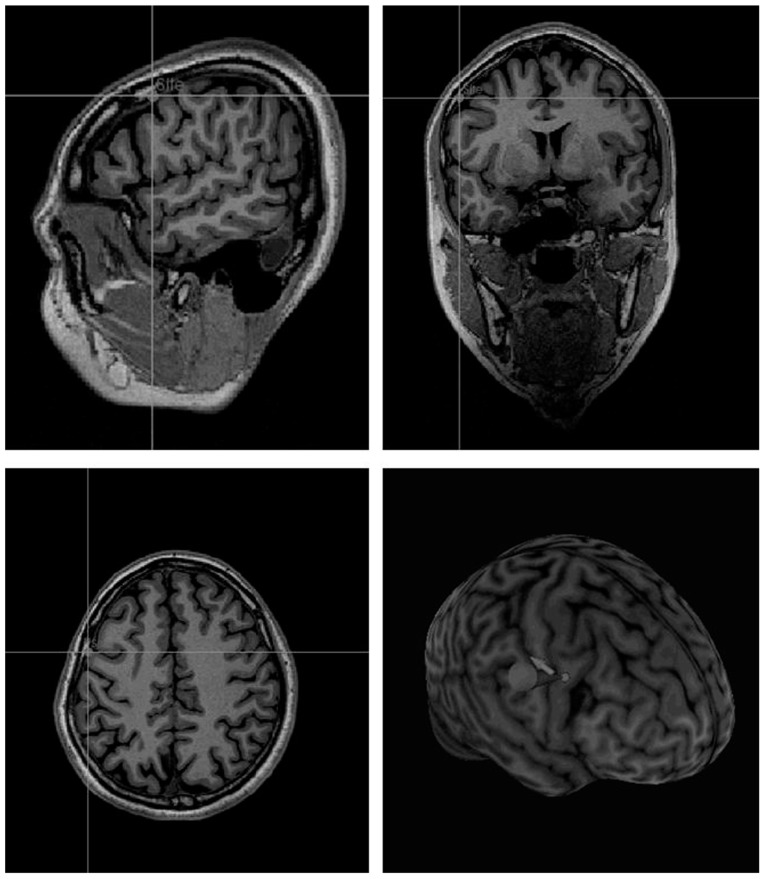
**Localization of the stimulation site on an individual MRI, according to theTalairach coordinates provided by Brown et al’s fMRI study (2008) (experimental group)**.

A Brainsight coregistration system (Rogue Research) was used to transform coordinates of the right PM larynx area to the individual MRI scan (**Figure 2**) and place the coil over the target site. The transformation as implemented in Brainsight is based on localization of the anterior and posterior commissures and then linear scaling in the corresponding axes. TMS was delivered via a figure-of-eight 50 mm coil and a Magstim Super Rapid Stimulator (Magstim, Whitland, UK). The coil was held anterior to the handle which was oriented parallel to the sagittal midline. Repetitive TMS was performed offline using the continuous theta-burst stimulation (cTBS) pattern (Di Lazzaro et al., 2005; Huang et al., 2005; Banissy et al., 2010) with three pulses of stimulation given at 50 Hz every 200 ms during 20 s, at 40% of machine output. This paradigm has been reported to reduce cortical excitability for 20–30 min after stimulation (Di Lazzaro et al., 2005; Huang et al., 2005). The stimulation intensity was based on that used successfully in previous studies (e.g., Kalla et al., 2009). This level is typically below motor threshold which is a reasonable rough guideline, although it should be remembered that motor threshold is not a guide for effective stimulation levels in other areas (see Stewart et al., 2001).

## RESULTS

**FIGURE 3:**
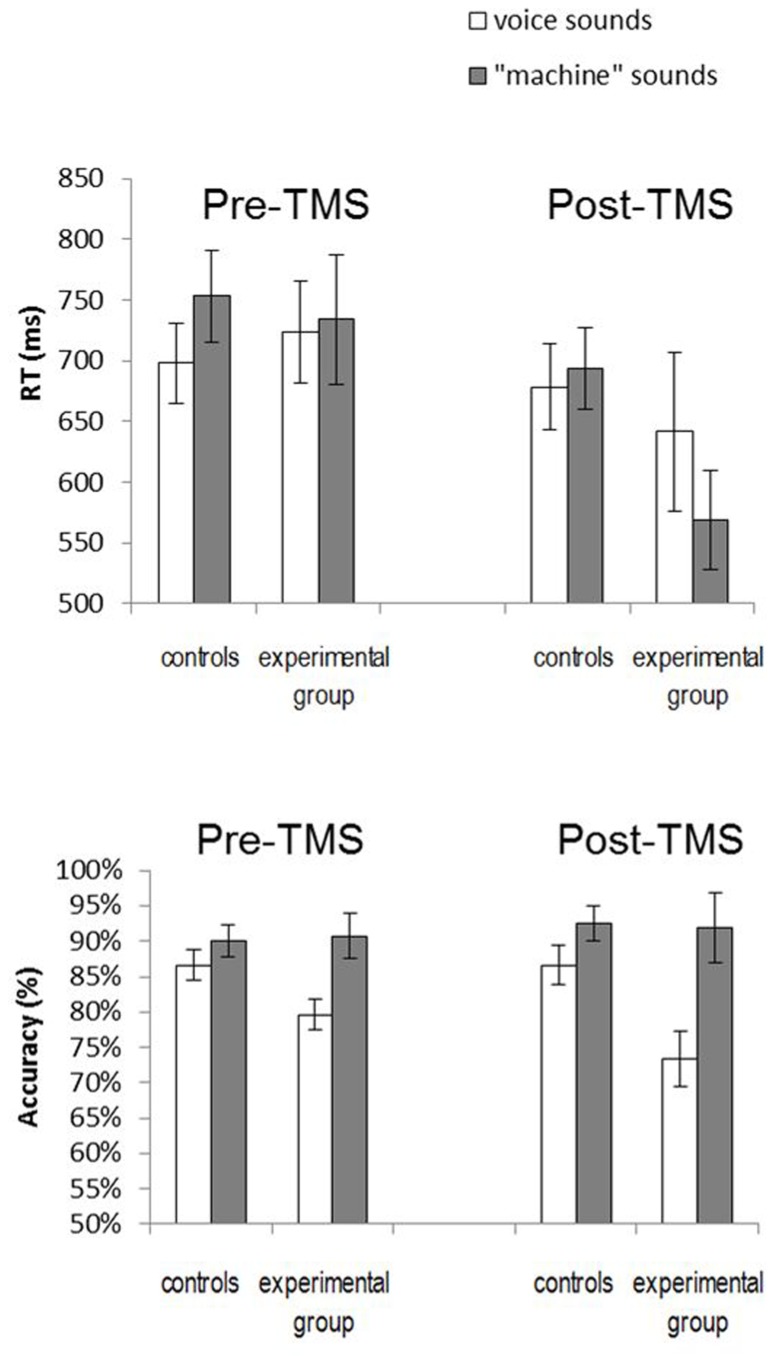
**Average reaction times (top) and performance accuracy (bottom) in the categorization task before and afterTMS for the group stimulated over the vertex (control) and over the larynx representation (experimental group)**.

Reaction times and performance data are illustrated in **Figure 3**. In order to gather a global view of our results we ran a three-way repeated measures analysis of variance (ANOVA) with a between-subjects Stimulation site factor (vertex vs PM larynx stimulation), a within-subjects Humanness factor (voice vs machine stimuli), and a within-subjects Session factor (before and after stimulation). Results showed a significant main effect of Humanness for accuracy (*F*(1,19) = 12.7, *p* = 0.002), with more errors for natural voice sounds, and a significant effect of Session for response times (*F*(1,19) = 7.7, *p* = 0.012) with overall faster responses after stimulation. Regarding RTs, the Humanness by Site and Session by Humanness interactions were also significant (*F*(1,19) = 6.1, *p* = 0.02; *F*(1,19) = 12.2, *p* = 0.002). Additionally, we ran a signal detection theory based analysis using the sensitivity index *d’* but no effect was significant, due to a large between-subject variance (all *p*-values > 0.1; interaction Session * Site: *F*(1,19) = 0.96, *p* = 0.33). Given this between-subject variance, we present below separate analyses for the pre- and the post-TMS results.

### BASELINE

Accuracy and RTs in the pre-TMS task served as a baseline measure to verify the lack of differences between control and experimental groups. This was confirmed via a two-way repeated measures ANOVA with a between-subjects Stimulation site factor (vertex vs PM larynx stimulation) and a within-subjects Humanness factor (number of correct responses for voice vs machine stimuli). While the main effect of Stimulation site and the interaction of Stimulation site and Humanness were not significant (*p* > 0.1), there was a significant Humanness effect (*F*(1,19) = 11.27; *p* = 0.003), with more categorization errors for voice compared to “machine” stimuli.

Reaction times were analyzed in a similar way by two-way repeated measures ANOVA with Stimulation site and Humanness factors (RTs for “voice” vs “machine” trials), using only correct trials and excluding response times that were more than 2.5 standard deviations from the mean in either direction. While the Stimulation site and Stimulation site * Humanness interaction were not significant (*F*(1,19) = 0.003; *p* = 0.95 and *F*(1,19) = 2.86; *p* = 0.11, respectively), there was a significant Humanness effect (*F*(1,19) = 5.96; *p* = 0.024) with faster categorization of voice stimuli. However, these faster responses are likely to be linked to the increase of errors for voice.

### POST-TMS RESULTS

The same analyses run on post-TMS accuracy showed no significant effects of the Stimulation site (*F*(1,19) = 1.78; *p* = 0.2). By contrast there was a significant main effect of Humanness (*F*(1,19) = 10.89; *p* = 0.003), with more errors on natural voice stimuli. Finally the Stimulation site * Humanness interaction was marginally significant (*F*(1,19) = 3.09; *p* = 0.095), with a poorer accuracy for categorization of natural voice sounds in the larynx-stimulated group.

The repeated measures ANOVA run on post-TMS RTs revealed a significant Humanness * Stimulation site interaction (*F*(1,19) = 5.75; *p* = 0.027). This was due to longer RTs for categorization of voice compared with “machine” stimuli in the larynx-stimulated group only (mean RT after larynx stimulation for voice: 641 ms, SEM = 65; for machine: 568 ms, SEM = 41; LSD *post hoc* test: *p* = 0.01, **Figure 2**. Note also that the mean RT in this group before stimulation for voice is 724 ms, SEM = 42, for machine: 734 ms, SEM = 54).

## DISCUSSION

In this study we tested the hypothesis that processing in motor areas has a causal role in voice perception. By using TMS to temporarily disrupt neural processing in a region of the right premotor cortex described as the larynx area (Brown et al., 2008), we observed a change in participants’ response times during a sound categorization task. TMS induced on the one hand a non-specific facilitation effect resulting in faster RTs, and on the other hand, an asymmetry in the RTs following voice and machine sounds. While human sounds were categorized faster than machine sounds before TMS, stimulation over the PM larynx area reversed this effect. Most importantly, this effect was accompanied by a reduction in accuracy for voice sounds, in the larynx-stimulated group only.

Faster baseline RT for human voice is in line with a previous behavioral study involving the same stimuli and the same task (Lévêque et al., 2012) and the study of Agus et al. (2010) demonstrating a voice processing advantage, not explained by purely spectral or purely temporal features of vocal sounds. This effect could reflect a bias toward biological sounds, which the listener is able to produce.

The facilitation effect after TMS may have different causes in the two groups. In the control group, the observed decrease of RT (-40 ms on average) is likely to be due to practice, as the task was repeated twice. By contrast, the larger reduction measured in the larynx-stimulated group (-124 ms on average) can be interpreted in terms of a cTBS effect on physiology. Although continuous TBS is more typically associated with an increase of RTs (Huang et al., 2005), some studies have reported a behavioral facilitation effect after cTBS or repetitive TMS, interpreted as an inter-hemispheric effect (Marzi et al., 1998; Huang et al., 2005; Fang et al., 2010). The hypothesis of an excitatory effect on the hemisphere contralateral to the stimulation site received strong support in Tracy et al.’s (2010) study combining repetitive TMS and fMRI. TMS was indeed found to enhance compensatory activation in homologous regions. Insofar as the hand and larynx representation are not far apart in the premotor/motor cortex, a contralateral (left hemisphere) “hyperfunction” may have facilitated motor responses given by the right hand.

The major finding of this paper is the differential effect of larynx area stimulation for vocal and non-vocal sounds. While we hypothesized that TMS over the motor cortex would increase RTs for vocal sounds, the significant effect we observed is the reduction of RTs for non-vocal sounds. One possible explanation to this unexpected effect may rely on the fact that, when the motor part of the auditory network is temporarily impaired, the task is performed uniquely on the basis of an auditory representation. This would imply that the transition via the motor system, while benefiting accuracy may actually have a cost in terms of processing time. A different interpretation could be that RTs are globally shorter due to the motor stimulation, and that voice processing is less shortened than machine sound processing, i.e., voice processing is impaired compared to machine sound processing. This interpretation is more consistent with the pattern of accuracy results. Indeed, accuracy was only affected by stimulation of the larynx site, with a selective decrease for voice sounds only. This decrease in accuracy together with an increase in RTs (relative to machine sounds) favors the hypothesis of a temporary perturbation of voice processing. Moreover, this interpretation is more in line with data reported in the literature. For instance, previous fMRI findings show sensorimotor interactions modulated by a producibility effect (Wilson and Iacoboni, 2006; Londei et al., 2010). There is also substantial evidence from previous studies to show that speech perception partly relies on motor representations (Iacoboni, 2008). Our study suggests that the premotor cortex may have a functional role in the perception of sung vocalizations. This is particularly interesting given that the stimuli were both brief (350 ms) and simple in their articulatory characteristics and the task did not involve working memory, which would have been likely to induce subvocal rehearsal. Our results are also in line with the recent TMS study of D’Ausilio et al. (2011), based on a similar working hypothesis about the involvement of the larynx area in vocal sound discrimination. However, this study of D’Ausilio et al. (2011) did not include any control sound, making it difficult to know whether the effect was specific to vocalizable sounds, or to sounds in general. In contrast, our study provides a comparison between TMS effect on voice and non-vocal sound perception and demonstrates a differential effect for the two timbres on categorization performance. To complete our understanding of the role of the larynx premotor area in voice perception, the question of lateralization remains to be addressed. To this aim, the effects of TMS on the right and left larynx representation should be compared within the same study.

Although we cannot draw any strong conclusion, the results of the current study suggest that cTBS of the right premotor larynx area may have a dual action on performance: a global facilitation effect, possibly due to proximity of the stimulated site with the hand area, and a relative impairment of voice compared to distorted voice processing. This relative impairment gives some support to the hypothesis that the right PM cortex has a functional role in voice perception, even in a perceptual framework that does not involve working memory. Furthermore, this result can be interpreted in terms of modulation of the coupling between production and perception systems by sound “producibility,” and more precisely voice “humanness.” Nonetheless, as a final note, we have to acknowledge that these results deserve further studies to disentangle the voice impairment vs the machine advantage hypotheses. These studies may want to use a within-subject approach to simplify the experimental design and reduce between-subject variance (here preventing the expected triple interaction Site by Session by Humanness).

## Conflict of Interest Statement

The authors declare that the research was conducted in the absence of any commercial or financial relationships that could be construed as a potential conflict of interest.

## Footnotes

1 These coordinates correspond to the more activated cluster in the right precentral gyrus during vocalization (dorsolateral cluster, “Larynx-Phonation Area”, Brown et al., 2008).
